# The Relationship Between Tramadol-Induced Oxidative Testis Injury and Reproductive Function Disorder and Adenosine Triphosphate

**DOI:** 10.3390/life15071078

**Published:** 2025-07-06

**Authors:** Fevzi Bedir, Hüseyin Kocatürk, Mehmet Sefa Altay, Renad Mammadov, Bahadır Süleyman, Taha Abdulkadir Coban, Gülce Naz Yazici, Seval Bulut, Halis Süleyman

**Affiliations:** 1 Department of Urology, Health Sciences University, Erzurum Regional Training and Research Hospital, Erzurum 25070, Turkey; kocaturk78@hotmail.com (H.K.); memsefaaltay@gmail.com (M.S.A.); 2 Department of Pharmacology, Faculty of Medicine, Erzincan Binali Yildirim University, Erzincan 24100, Turkey; renad_mamedov@hotmail.com (R.M.); bahadirsuleyman@yandex.com (B.S.); sevalbulut2010@hotmail.com (S.B.); halis.suleyman@gmail.com (H.S.); 3 Department of Biochemistry, Faculty of Medicine, Erzincan Binali Yildirim University, Erzincan 24100, Turkey; akcoban@gmail.com; 4 Department of Histology and Embryology, School of Medicine, Erzincan Binali Yildirim University, Erzincan 24100, Turkey; gulcenazyazici.ank@gmail.com

**Keywords:** antioxidant, ATP, infertility, pro-inflammatory, rat, tramadol

## Abstract

Tramadol, a central analgesic drug, is used to treat moderate to severe pain but can cause reproductive disorders. The pathogenesis of tramadol-induced reproductive damage may involve increased oxidative stress, pro-inflammatory cytokines, ATP depletion, and reduced antioxidant levels. In this study, subjects were divided into four groups: healthy control (HC), tramadol only (TM), ATP only (ATP), and ATP + tramadol (ATM). ATP was administered intraperitoneally at 4 mg/kg, and tramadol was administered orally at 50 mg/kg. Distilled water was given to the HC group. This regimen was repeated for three weeks. At the end of the treatment, testicular tissues from six rats in each group were analyzed biochemically and histopathologically after euthanasia. The remaining rats’ reproductive functions were evaluated. Long-term tramadol exposure resulted in oxidative stress, inflammation in testicular tissue, and reduced male reproductive capacity. Thinning of seminiferous tubule walls and thickening of basement membrane, irregularity in germ cells, increase in interstitial connective tissue, congestion in vessels, increase in Leyding cells and hyperplasia were found in the TM group. ATP treatment significantly reduced tramadol-induced increases in oxidants and pro-inflammatory cytokines, reversed the decline in antioxidants, and mitigated infertility in testicular tissue. Furthermore, ATP preserved the morphology of the testicular tissue. These findings suggest that ATP may offer therapeutic potential for tramadol-induced infertility.

## 1. Introduction

Tramadol is a centrally-acting analgesic that is metabolized into more potent metabolites through the O-demethylation pathway [1]. Its analgesic effect is mediated through the stimulation of μ-opioid receptors [2]. In the central nervous system, tramadol also binds to µ- and δ- opioid receptors, inhibiting norepinephrine and serotonin reuptake, while directly stimulating serotonin release [1]. Tramadol is currently used as an analgesic in the treatment of moderate and severe pain [3]. However, even at analgesic doses, it can induce oxidative and inflammatory damage in various organs and tissues [4]. Furthermore, tramadol can lead to decreased sexual activity and even reproductive dysfunction [5]. Preclinical studies have demonstrated that tramadol can impair the reproductive organs in both male and female animals [6]. Increased levels of oxidants and pro-inflammatory cytokines, including interleukin-1β (IL-1β), IL-6, tumor necrosis factor-α (TNF-α), and nuclear factor-kappa B (NF-κB), coupled with decreased antioxidant levels, have been implicated in the pathogenesis of tramadol-induced reproductive organ damage and dysfunction [7]. Mousavi et al. [8] reported a correlation between increased intracellular reactive oxygen species (ROS) production and decreased antioxidant and ATP levels. Faria et al. [9] demonstrated that tramadol induces cellular oxidative damage, leading to intracellular ATP depletion. Elmorsy et al. also reported that tramadol significantly decreased the activities of mitochondrial complexes I and III, with a parallel decrease in ATP production [10]. It has been emphasised in the literature that tramadol causes oxidative stress, inflammation, mitochondrial respiratory enzyme dysfunction, mitochondrial membrane potential dysfunction and consequently ATP synthesis inhibition [11]. These findings suggest that tramadol-induced reproductive organ damage and dysfunction may be associated with intracellular ATP depletion. Moreover, they suggest that ATP supplementation may have therapeutic potential in tramadol-induced reproductive organ damage and dysfunction.

ATP, which was examined in this study for its potential protective effects against tramadol-induced testicular damage and reproductive dysfunction in rats, is a nucleoside triphosphate composed of adenine, a ribose sugar, and three phosphate groups [12]. ATP levels have been shown to be critical in regulating essential cellular processes such as growth, development, and survival in both in vivo and in vitro studies [13]. Furthermore, it is known to be involved in the synthesis of ROS-scavenging antioxidants [14]. ATP has also been shown to provide energy for the synthesis of low molecular weight antioxidants [15]. Mitochondrial ATP production is necessary during the sperm’s journey from the uterus to the fallopian tube to reach the oocyte. ATP is produced by oxidative phosphorylation. In addition to ATP, potentially harmful ROS are also produced in the mitochondria as by-products in this process, including impairing sperm quality [16]. We found no previous studies investigating the effect of ATP on tramadol-induced testicular damage and reproductive dysfunction. Therefore, this study aimed to investigate the effects of tramadol on testicular damage and reproductive function in rats, as well as the potential protective effects of ATP against this damage. Additionally, we sought to evaluate the relationship between exogenous ATP administration and the production of oxidants, antioxidants, and pro-inflammatory cytokines.

## 2. Material and Methods

### 2.1. Animals

Forty-eight albino male and seventy-two female Wistar rats, weighing 280–290 g (10–12 weeks), were obtained from the Binali Yıldırım University Medical Experimental Application and Research Center, Türkiye. Before the experiment, the rats were divided into four equal groups and housed under appropriate conditions at a controlled room temperature of 22 °C with a 12-h light/dark cycle. They were provided with *ad libitum* access to tap water and standard laboratory chow (experimental animal feed; *Bayramoglu* AS, Erzurum, Türkiye). The experimental protocols and procedures were approved by the local Animal Experimentation Ethics Committee (approval date: 25 January 2024; meeting number: 01/45). All procedures were conducted in accordance with the guidelines and regulations of the Medical Experimental Application and Research Center’s Animal Experiments Ethics Committee.

### 2.2. Chemical Substances

The sodium thiopental used in this study was provided by IE Ulagay (İstanbul, Türkiye), ATP by Zdorove Narodu (Kharkiv, Ukraine), and tramadol by Hel Abdi İbrahim İlaç (İstanbul, Türkiye).

### 2.3. Experimental Groups

The animals were divided into four groups: a control group (HC), a tramadol-only group (TM), an ATP-only group (ATP), and an ATP + tramadol group (ATM).

### 2.4. Experimental Procedure

The animals in the ATM (n = 12) and ATP (n = 12) groups received intraperitoneal injections of ATP at a dose of 4 mg/kg [17]. The HC (n = 12) and TM (n = 12) groups received intraperitoneal injections of distilled water as a vehicle. One hour after the ATP and distilled water injections, the ATM and TM groups were administered 50 mg/kg of tramadol by oral gavage [18]. This procedure was repeated daily for three weeks. At the end of the three-week period, six animals from each group were euthanized with a lethal dose of sodium thiopental (50 mg/kg), and their testicular tissues were collected. Levels of malondialdehyde (MDA), total glutathione (tGSH), superoxide dismutase (SOD), catalase (CAT), IL-18, IL-6, and tumor necrosis factor-alpha (TNF-α) were measured in the testicular tissues. The tissues were also examined histopathologically. The remaining six animals from each group were used to evaluate reproductive function. Each male rat was housed in a separate cage, and three female rats were added to each cage. Males that failed to impregnate female rats during the two-month period were considered infertile. The results from all experimental groups were then compared.

### 2.5. Specimen Preparation

The testicular tissues were first washed with physiological saline, then pulverized under liquid nitrogen, and homogenized. The supernatants were then used to measure MDA, GSH, SOD, and CAT levels.

### 2.6. MDA, GSH, SOD, and CAT Analyses in Testicular Tissues

Tissue SOD, GSH, and MDA levels were measured using commercially available rat specific ELISA kits (Cayman Chemical Company; Ann Arbor, MI, USA; product numbers 706002, 703002, and 10009055, respectively), following the manufacturer’s instructions. The 96-well ELISA microplate provided in the kit is coated with the antibody of the parameter of interest. Standards and samples are added to these wells. Then, a specific biotinylated detection antibody and streptavidin-HRP conjugate are added to the microplates for incubation. The substrate solution is then added to each well. The enzyme-substrate reaction is terminated using acidic stop solution, and the colour formed is measured spectrophotometrically. CAT activity was measured using the method described by Goth [19].

### 2.7. TNF-α, IL-1β, and IL-6 Analyses in Testicular Tissues

The samples were weighed, and any excess tissue was trimmed away. The testicular tissue samples were snap-frozen in liquid nitrogen and homogenized with a mortar and pestle. The samples were then stored at −80 °C until use. Phosphate-buffered saline (PBS, pH 7.4) was added at a ratio of 1:10 (*w*/*v*). The samples were vortexed for 10 s and centrifuged at 10,000 × g for 20 min. The supernatant was collected and stored at −80 °C. Levels of TNF-α (ng/L), IL-1β (pg/L), and IL-6 (ng/L) were measured using commercially available ELISA kits (Eastbiopharm Co., Ltd., Hangzhou, China). To determine these levels, the kits use a double antibody sandwich enzyme-linked immunosorbent assay (ELISA). Absorbance was read at 450 nm using a plate reader.

### 2.8. Histopathological Examination

The tissue samples were fixed in 10% formalin for histological analysis. The tissue samples were washed in running tap water overnight. The tissues were dehydrated through a series of graded ethanol solutions (70, 80, 90, and 100%). The tissues were then cleared in xylene and embedded in paraffin. Sections (4–5 μm thick) were cut from the paraffin blocks and stained with hematoxylin and eosin (H&E). Images were captured using Olympus DP2-SAL imaging software (Olympus, Tokyo, Japan). A blinded histopathologist performed the histopathological analysis. The severity of damage to testicular tissues was calculated using the grading system recommended by Cosentino et al. (Grade 1: Normal testicular architecture with an orderly arrangement of germinal cells; Grade 2: Injury showed less orderly, non-cohesive germinal cells and closely packed seminiferous tubules; Grade 3: Injury exhibited disordered sloughed germinal cells, with reduced size of pyknotic nuclei and less distinct seminiferous tubule borders; Grade 4: Injury exhibited seminiferous tubules that were closely packed with coagulative necrosis of the germinal cells) [20]. A different score was assigned to each testicular area, and the final Cosentino’s grade for each testis was calculated by multiplying the grade of each area by the percentage of the total surface that it occupied.

### 2.9. Statistical Analyses

The results are presented as mean ± standard deviation (SD). Levene’s test indicated that the data were normally distributed, and the Shapiro-Wilk test confirmed homogeneity of variance. Therefore, one-way ANOVA was used for statistical analysis. Post hoc analysis was performed using Tukey’s HSD test. Histopathological analysis was performed by the Kruskal–Wallis test followed by post hoc Dunn’s test. All statistical analyses were performed using SPSS software (version 22.0), Armonk, NY, USA: IBM Corp., and *p* < 0.05 was considered statistically significant.

## 3. Results

### 3.1. Testicular Tissue MDA and GSH Analysis Results

**Figure 1:**
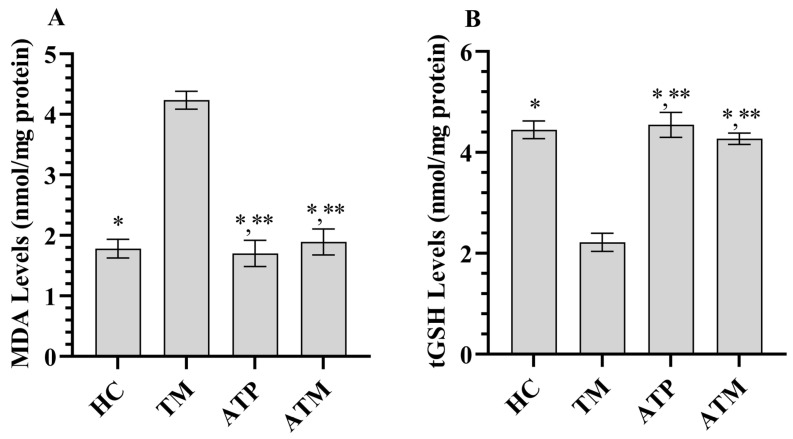
(**A**) MDA levels in testicular tissue, (**B**) tGSH levels in testicular tissue *; *p* < 0.001 vs. TM, **; *p* > 0.05 vs. (n = 6) HC. MDA; malondialdehyde, tGSH; total glutathione, HC; healthy group, TM; tramadol group, ATP; ATP group, ATM; ATP + tramadol group.

**Figure 2:**
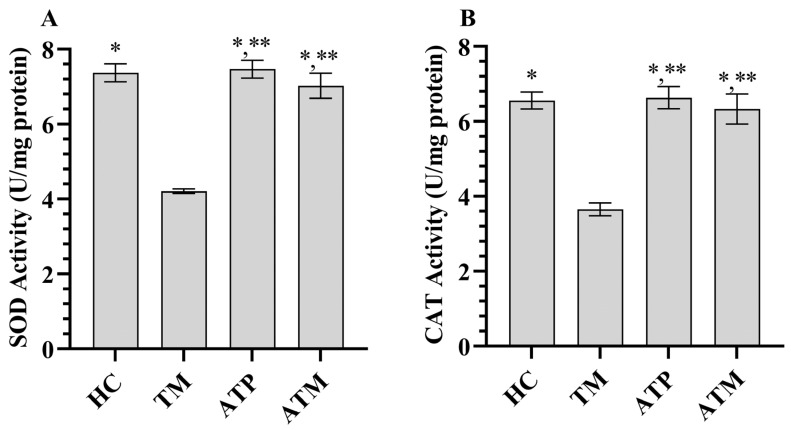
(**A**) SOD activities in the testicular tissues, (**B**) CAT activities in the testicular tissues; *; *p* < 0.001 vs. TM, **; *p* > 0.05 vs. (n = 6) HC. SOD; superoxide dismutase, CAT; catalase, HC; healthy group, TM; tramadol group, ATP; ATP group, ATM; ATP + tramadol group.

As shown in Figure 1, tramadol treatment increased MDA levels and decreased tGSH levels in testicular tissue (*p* < 0.001). ATP administration alone did not significantly affect MDA or tGSH levels in testicular tissue (*p* > 0.05). However, ATP significantly attenuated the tramadol-induced increase in MDA and decrease in tGSH (Table 1; *p* < 0.001). As shown in Figure 2, tramadol treatment decreased SOD and CAT activities in testicular tissue (*p* < 0.001). ATP administration alone did not significantly affect SOD or CAT activities in testicular tissue (*p* > 0.05); however, ATP significantly attenuated the tramadol-induced decrease in SOD and CAT activities (Table 1; *p* < 0.001).

### 3.2. Testicular Tissue IL-1β, IL-6, and TNF-α Analysis Results

**Figure 3:**
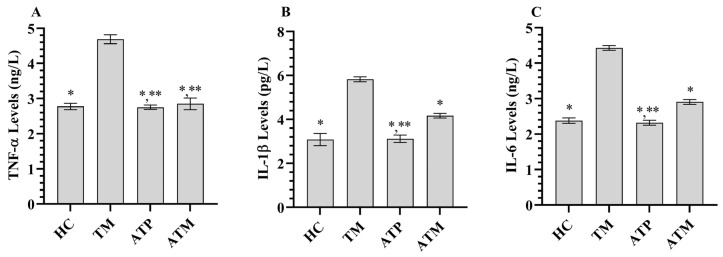
(**A**) TNF-α levels in the testicular tissues, (**B**) IL-1β levels in the testicular tissues, (**C**) IL-6 levels in the testicular tissues; *; *p* < 0.001 vs. TM, **; *p* > 0.05 vs. (n = 6) HC. TNF-α; tumor necrosis factor α, IL-1β; interleukin-1β, IL-6; interleukin-6, HC; healthy group, TM; tramadol group, ATP; ATP group, ATM; ATP + tramadol group.

Tramadol treatment significantly increased the levels of pro-inflammatory cytokines IL-1β, IL-6, and TNF-α in testicular tissue (*p* < 0.001). ATP administration alone did not significantly affect IL-1β, IL-6, or TNF-α levels in testicular tissue. However, ATP significantly attenuated the tramadol-induced increase in IL-1β, IL-6, and TNF-α levels (Figure 3 and Table 1; *p* < 0.001).

### 3.3. Reproduction Test Results

As shown in Table 2, no infertility was observed in the HC and ATP groups. In the TM group, 4 of 6 rats were infertile (66.7%). On the other hand, in rats receiving ATP treatment with tramadol, infertility was detected in 1 of 6 rats (16.7%).

### 3.4. Histopathological Findings

According to Cosentino scoring, tramadol caused significant deterioration in the histological structure of the testis (*p* < 0.001). The severity of damage was found to be significantly lower in the testes of rats receiving tramadol and ATP treatment (*p* < 0.001).

**Figure 4:**
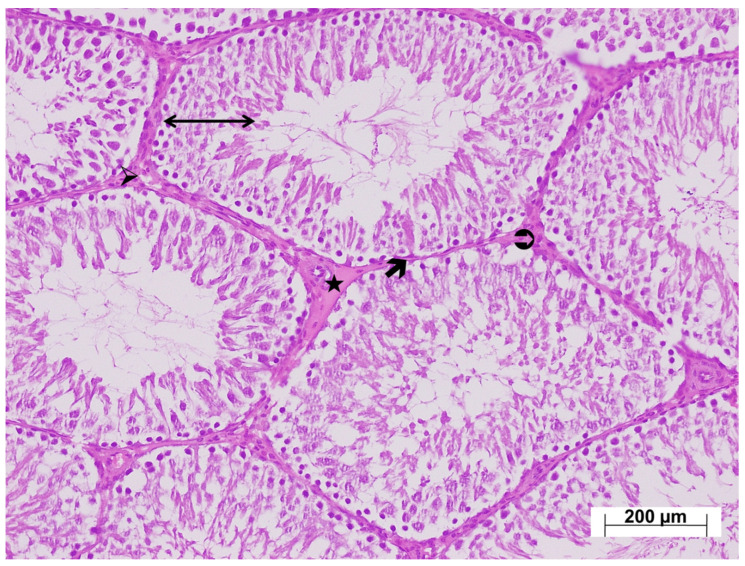
H&E-stained testicular tissues from the healthy group exhibiting a normal histological appearance; ↔: seminiferous tubule wall, 🡲: basement membrane, ➢: interstitial connective tissue, ⮊: Leydig cells, ★: vessels, ×100.

**Figure 5:**
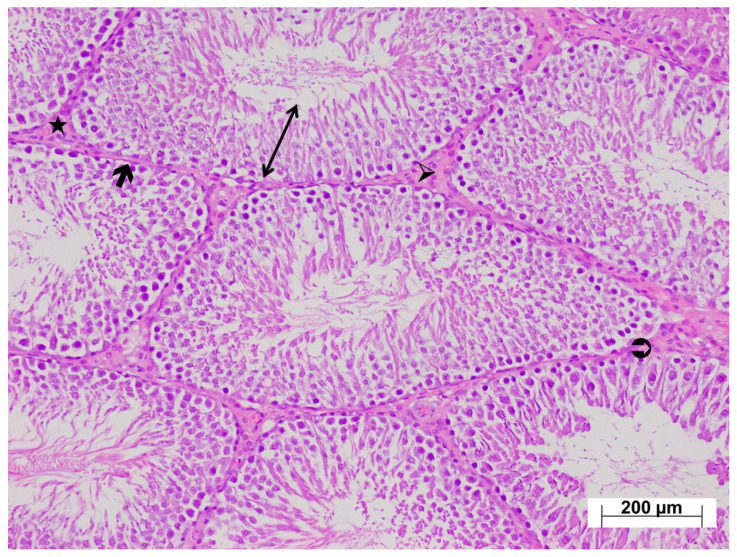
H&E-stained testicular tissues from the ATP group exhibiting a normal histological appearance; ↔: seminiferous tubule wall, 🡲: basement membrane, ➢: interstitial connective tissue, ⮊: Leydig cells, ★: vessel, ×100.

As shown in Figure 4, examination of testicular tissues from the HC group revealed normal seminiferous tubule size and morphology, with germinal cell lines normally arranged and aligned regularly along the basement membrane. Basement membrane thickness was normal, and the interstitial connective tissue, vessels, and Leydig cells within the interstitial tissue were naturally aligned. The testicular tissue from the ATP-only group exhibited normal histological architecture (Figure 5).

**Figure 6:**
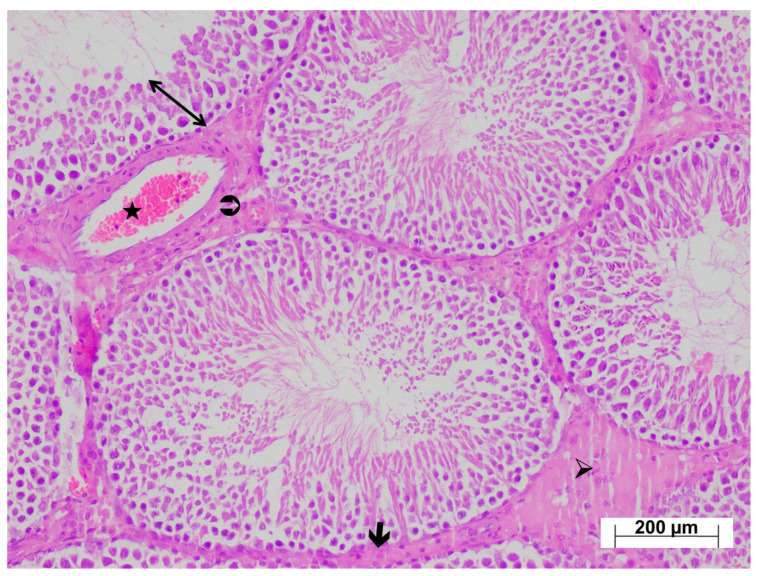
Low magnification H&E-stained testicular tissue from the TM group exhibiting ↔: an irregular seminiferous tubule wall, 🡲: thickened basement membrane, ➢: markedly increased interstitial connective tissue, ⮊: densely distributed hyperplastic Leydig cells, ★: intensely congested vessels, ×100.

**Figure 7:**
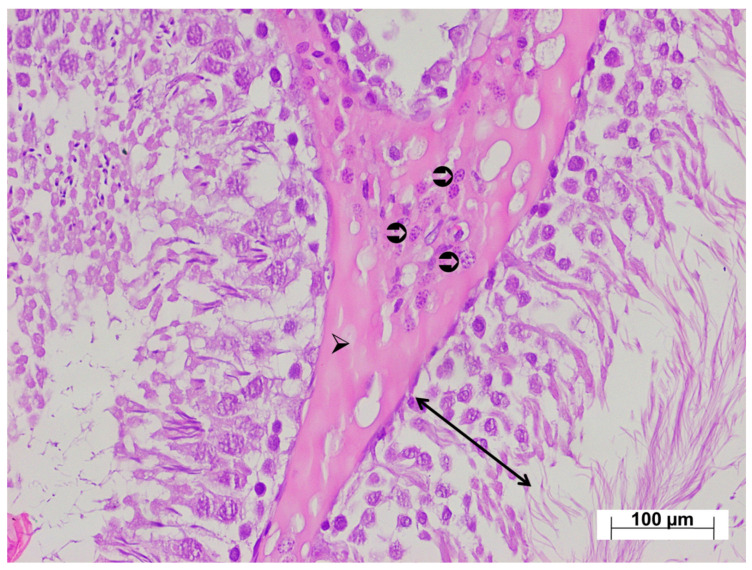
High magnification H&E-stained testicular tissue from the TM group exhibiting; ↔: an irregular seminiferous tubule wall, ➢: markedly increased interstitial connective tissue, ⮊: densely distributed Leydig cells, ×200.

**Figure 8:**
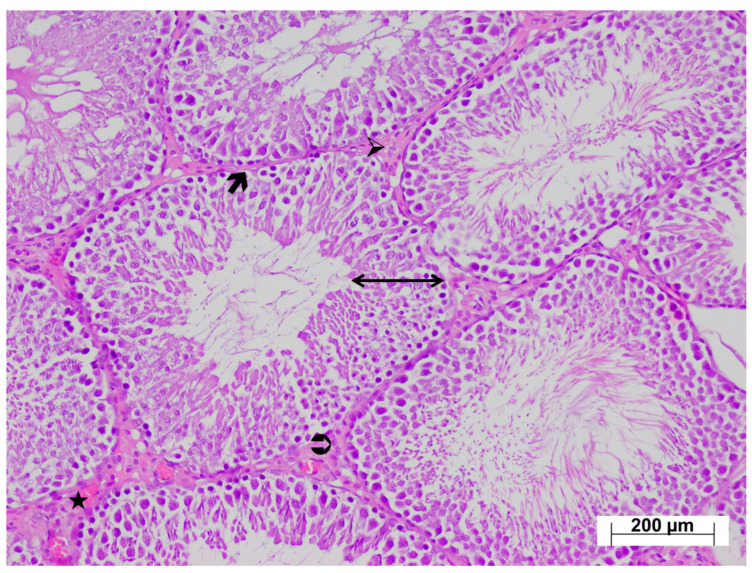
H&E-stained testicular tissues from the ATM group exhibiting; ↔: a normal seminiferous tubule wall, 🡲: mildly thickened basement membrane, ➢: increased interstitial connective tissue, ⮊: normal-appearing Leydig cells, ★: mildly congested vessels, ×100.

Low-magnification examination of testicular tissues from the tramadol-treated group revealed relatively thinner seminiferous tubule walls compared with the control group, and impaired arrangement of developing germ cells. Marked thickening of the seminiferous tubule basement membranes was observed. The interstitial connective tissue was markedly thicker than in the control group, with intense vascular congestion (Figure 6). High-magnification examination revealed Leydig cell hyperplasia within the thickened interstitial connective tissue, with increased numbers of Leydig cells arranged in layers (Figure 7). In the testicular tissues from the ATM group, both the walls of the seminiferous tubules and germ cell distribution appeared normal. The basement membranes of the seminiferous tubules were thinner compared with the injury group, and the interstitial connective tissue was also thinner. Leydig cell density was also lower than in the injury group. Occasional Leydig cell hyperplasia was observed, along with mild vascular congestion (Figure 8).

## 4. Discussion

This study investigated the effects of tramadol on testicular damage and reproductive function in rats. It also examined the potential protective effects of ATP against tramadol-induced testicular damage and reproductive dysfunction. The literature on the intracellular and extracellular effects of exogenous ATP therapy is confusing [21]. Contrary to the view that ATP cannot enter the cell, in the 1970s, labeled ATP was shown to enter muscle cells. On the other hand, the rapid degradation of extracellular ATP has been a concern for ATP therapy. However, it has been argued that its breakdown product, AMP, mediates the protective effects of ATP [22]. The results showed that 66.7% of the tramadol-treated rats developed infertility. This is consistent with previous studies reporting that tramadol can lead to decreased sexual activity and even reproductive dysfunction [5]. Increased levels of pro-inflammatory cytokines, including oxidants, IL-1β, IL-6, TNF-α, and NF-κB, as well as decreased antioxidant levels, have been implicated in the pathogenesis of tramadol-induced reproductive organ damage and dysfunction in previous studies [7]. Therefore, in this study, levels of oxidants, antioxidants, and pro-inflammatory cytokines were measured in testicular tissue from tramadol-treated animals, and their association with reproductive dysfunction was evaluated. Oxidative tissue damage is associated with increased oxidant levels and decreased antioxidant levels [23,24]. A previous study also used oxidant and antioxidant parameters, including MDA, GSH, and SOD, to evaluate oxidative testicular injury [23]. In this study, MDA levels were measured to determine whether tramadol induces oxidative stress in testicular tissue. MDA levels were higher in the tramadol-treated group than in the control and ATP-treated groups. MDA, the end product of lipid peroxidation, is an indicator of increased ROS levels [25]. Yapanoglu et al. reported higher MDA levels in damaged testicular tissue than in healthy and treated groups [26].

Our findings are consistent with previous studies demonstrating the involvement of increased oxidants and decreased antioxidants in the pathogenesis of tramadol-induced reproductive organ damage and dysfunction [7].

Studies have shown that tramadol disrupts the balance between ROS and antioxidants by reducing antioxidant levels [27]. Therefore, we measured tGSH, SOD, and CAT levels to investigate the mechanisms underlying tramadol-induced testicular tissue injury and reproductive dysfunction in rats. Excess ROS, which can disrupt tissue morphology and physiology, are neutralized by endogenous enzymatic and non-enzymatic antioxidant defense systems, including SOD, CAT, and GSH [28]. However, when antioxidant capacity is insufficient to neutralize ROS, oxidative stress occurs in tissues and organs [24].

We observed a marked decrease in the levels of these antioxidants in the testicular tissues of tramadol-treated animals. Previous studies have reported decreased GSH levels along with parallel significant decreases in SOD and CAT levels in damaged tissues [29]. Furthermore, antioxidant supplementation is recommended to protect tissues and organs against oxidative damage [24]. Inflammation and oxidative stress are inextricably linked in the male reproductive system. Inflammation and oxidative stress can impair reproductive function, with each potentially exacerbating the other [30]. Long-term tramadol use can also cause organ damage by inducing oxidative stress and the release of pro-inflammatory factors [27]. Increased levels of oxidants and pro-inflammatory cytokines, including IL-1β, IL-6, TNF-α, and NF-κB, have been shown to contribute to reproductive organ damage and dysfunction in both sexes. A positive correlation has also been observed between increased pro-inflammatory cytokine production and oxidant levels [31]. MDA is a product of ROS. Increased ROS production activates NF-κB and induces TNF-α and IL-1β gene transcription [32]. Consistent with previous findings, our results indicate that pro-inflammatory cytokines, along with oxidants, play an important role in the pathogenesis of tramadol-induced reproductive organ damage and dysfunction. As mentioned earlier, increased levels of oxidants, IL-1β, IL-6, TNF-α, and NF-κB have been implicated in tramadol-induced reproductive organ damage and dysfunction [7].

Previous studies have shown a link between increased intracellular ROS production and antioxidant and ATP depletion [8]. Another study highlighted that tramadol-induced cellular oxidative damage also leads to intracellular ATP depletion [9]. These findings suggest that tramadol-induced reproductive organ damage and dysfunction may be associated with intracellular ATP depletion and potentially treatable with ATP. Our results indicate that ATP prevented tramadol from altering oxidant, pro-inflammatory cytokine, and antioxidant levels in testicular tissue, and reduced infertility rates in rats. These findings are consistent with previous studies reporting that ATP plays a role in ROS scavenging and antioxidant synthesis, and serves as an energy source for these processes [14,15]. Furthermore, ATP has been shown to inhibit oxidant and pro-inflammatory cytokine overproduction and to protect female reproductive organs [17].

However, our literature search did not yield any information regarding the potential of extracellular ATP to increase inflammatory responses in the testes or to adversely affect peritubular cells [33]. Another study reported that ROS increased pro-inflammatory cytokine production and decreased antioxidant levels, leading to decreased ATP concentrations in the testes [34].

Analysis showed a positive correlation between our biochemical and histopathological findings. Severe histopathological changes, including thinning of the seminiferous tubule walls, thickening of the tubular basement membrane, impaired arrangement of developing germ cells, increased interstitial connective tissue, intense vascular congestion, and Leydig cell hyperplasia, were observed in the testes of tramadol-treated animals. Recent studies have also shown that tramadol can cause severe testicular damage [29]. Tramadol can also lead to structural impairments, including decreased spermatogenesis, seminiferous tubule degeneration, decreased spermatogenic cell numbers, and Leydig cell depletion [35]. It has also been reported to cause vascular bleeding, interstitial vacuoles, germ cell exfoliation, chromatin degeneration in elongated spermatids, and testicular malformations in adult and adolescent rats [36]. However, we found no previous evidence suggesting that tramadol induces Leydig cell hyperplasia. In this study, ATP attenuated the toxic effects of tramadol on the testes, suggesting that tramadol may induce oxidative and inflammatory damage by decreasing ATP levels in testicular tissue. One study supporting this hypothesis reported decreased sperm counts, impaired morphology and viability, and reduced seminiferous tubule diameter and epithelial thickness in cases of low ATP [34]. Measuring ATP levels in the testicular tissue of tramadol-treated animals is important for understanding the mechanisms underlying tramadol-induced injury.

Tramadol is widely used worldwide as a painkiller, but it is also a drug that is abused. It has been noted in the literature that it can reduce semen quality in the human male reproductive system, increase oxidative damage in sperm, and impair male fertility by decreasing antioxidants in testicular tissues [37]. Considering its widespread use, strategies for the protection of the male reproductive system become even more important. Our study findings provide evidence that the addition of concomitant ATP therapy to tramadol use protects testicular tissue from oxidative and inflammatory damage and supports fertility.

Today, environmental pollution has shown negative effects on reproductive health. Studies have shown that heavy metals, in particular, cause serious damage to sperm DNA, damage the cell at molecular and cellular levels, and as a result, negatively affect fertility [38].

This study has certain limitations that should be acknowledged. Firstly, although several oxidative stress and antioxidant parameters were evaluated, a comprehensive assessment of the overall oxidative and antioxidant status was not performed. This limits the ability to fully interpret the dynamic balance between pro-oxidant and antioxidant systems across the experimental groups. Additionally, the activities of key antioxidant enzymes, including glutathione peroxidase and glutathione reductase, could not be measured, which restricts the mechanistic understanding of the antioxidant defense system. Finally, the experimental protocol focused on a short-term exposure period, and thus, long-term or cumulative effects could not be assessed. Future studies should address these limitations by incorporating a broader range of oxidative stress markers, measuring ATP levels directly, increasing sample size, and extending the duration of follow-up to strengthen the validity and applicability of the results.

## 5. Conclusions

Long-term tramadol exposure increased oxidant and pro-inflammatory markers while decreasing antioxidant markers and impairing male reproductive capacity in testicular tissue. Tramadol also disrupted normal testicular tissue morphology. Tramadol caused thinning of seminiferous tubule walls and thickening of basement membrane, irregularity in germ cells, increase in interstitial connective tissue, congestion in vessels, increase in Leyding cells and hyperplasia. ATP administration alone did not affect testicular tissue biochemistry, morphology, or male reproductive capacity. Furthermore, ATP significantly prevented tramadol-induced increases in oxidant and pro-inflammatory cytokine levels, decreases in testicular tissue antioxidant levels, infertility and histopathological changes. Our results suggest that ATP may be a useful treatment for male reproductive organ damage and dysfunction associated with long-term tramadol use. These study results may be encouraging for clinical trials to test whether co-administration of ATP with tramadol would be effective in preventing or reducing any possible damage to testicular structures.

## Figures and Tables

**Table 1:** Oxidant, antioxidant and pro-inflammatory analysis results in testicular tissues.

Biochemical Parameters	HC	TM	ATP	ATM	F (3, 20)	*p* Values
	Mean ± Standard Deviation					
MDA	1.78 ± 0.15 *	4.23 ± 0.15	1.70 ± 0.22 *^,^**	1.89 ± 0.22 *^,^**	258.099	<0.001
tGSH	4.45 ± 0.18 *	2.22 ± 0.18	4.56 ± 0.25 *^,^**	4.27 ± 0.11 *^,^**	215.054	<0.001
SOD	7.37 ± 0.24 *	4.21 ± 0.06	7.47 ± 0.24 *^,^**	7.03 ± 0.33 *^,^**	252.414	<0.001
CAT	6.56 ± 0.23 *	3.65 ± 0.17	6.63 ± 0.30 *^,^**	6.33 ± 0.40 *^,^**	149.396	<0.001
TNF-α	2.78 ± 0.09 *	4.69 ± 0.13	2.75 ± 0.06 *^,^**	2.85 ± 0.16 *^,^**	395.830	<0.001
IL-1β	3.08 ± 0.28 *	5.83 ± 0.11	3.12 ± 0.17 *^,^**	4.17 ± 0.11 *	298.600	<0.001
IL-6	2.38 ± 0.19 *	4.43 ± 0.17	2.32 ± 0.18 *^,^**	2.91 ± 0.17 *	192.944	<0.001

*; *p* < 0.001 vs. TM, **; *p* > 0.05 vs. (n = 6) HC. MDA; malondialdehyde, tGSH; total glutathione, SOD; superoxide dismutase, CAT; catalase, TNF-α; tumor necrosis factor α, IL-1β; interleukin-1β, IL-6; interleukin-6, HC; healthy control group, TM; tramadol group, ATP; ATP group, ATM; ATP + tramadol group. Statistical analysis was performed with one-way ANOVA-Tukey HSD.

**Table 2:** The study groups’ fertility results.

Groups	Number of Animals	Developing Infertility	%	Not Developing Infertility	%
**HC**	**6**	**0**	**0**	**6**	**100**
**TM**	**6**	**4**	**66.7**	**2**	**33.3**
**ATP**	**6**	**0**	**0**	**6**	**100**
**ATM**	**6**	**1**	**16.7**	**5**	**83.3**

## Data Availability

The datasets used and/or analysed during the current study are available from the corresponding author on reasonable request.
